# Three-dimensional cellular construct with impregnated silicon nanowires for intracellular optoelectronic biointerface

**DOI:** 10.1016/j.mtbio.2025.102039

**Published:** 2025-07-02

**Authors:** Nadi Hathot, Tania Assaf, Layan Habib, Noa M. Cohen, Dana Nir, Alexander Borodetsky, Shiri Karni-Ashkenazi, Menahem Y. Rotenberg

**Affiliations:** a Department of Biomedical Engineering, Technion- Israel Institute of Technology, Haifa, 32000, Israel; b Russell Berrie Nanotechnology Institute, Technion - Israel Institute of Technology, Haifa, 32000, Israel

## Abstract

Three-dimensional tissue models are considered a more comprehensive replica of the in vivo microenvironment than their traditional monolayer counterparts. Therefore, tissue engineering methods have the potential to revolutionize biomedical research by allowing researchers to shift away from animal models while improving model relevance. However, while state-of-the-art electrical devices can perturb the biophysical cell niche in 2D monolayers, the biomodulation “toolkit” available for 3D application does not meet the required level of complexity, specificity, and accuracy, limiting the ability to perform intracellular electrical modulation of cells inside 3D cellular constructs. In this work, a 3D e-scaffold impregnated with free-standing silicon nanowires was developed to enable local and leadless optoelectronic modulation at subcellular resolution. The versatility, simplicity, and biocompatibility of e-scaffolds, comprised of alginate and/or collagen, were demonstrated with a fibroblast cell line and primary cardiac cells. Their utility for bioelectrical modulation was demonstrated by optically stimulating intracellular nanowires and visualizing the calcium response using confocal microscopy. The e-scaffold was used to study the coupling between cardiac myofibroblasts and cardiomyocytes in a 3D context. The e-scaffold was found to enable straightforward 3D tissue culture as well as intracellular electrical modulation at subcellular resolution.

## Graphical abstract

**Figure:**
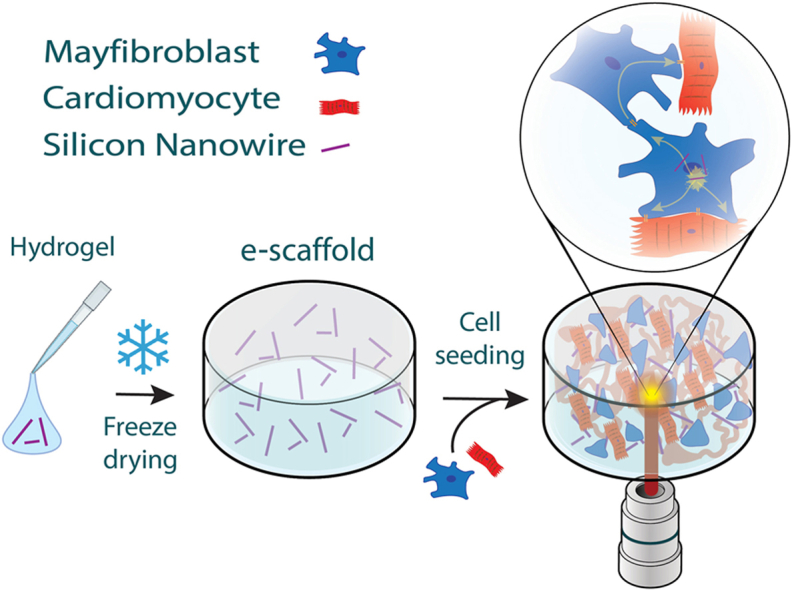

## 1 Introduction

The cellular microenvironment contains a well-orchestrated and tightly regulated combination of biological, biochemical, biomechanical, and bioelectrical cues. While most biomedical research is conducted on in vitro two-dimensional (2D) monolayers of cells, it has become clear that three-dimensional (3D) tissue cultures are a much more comprehensive representation of the in vivo cellular microenvironment [1]. However, controlling the cellular niche, especially in the context of bioelectrical cues, is much more challenging in 3D tissue cultures than with traditional 2D monolayers. For instance, bioelectronics with subcellular resolution, such as patch pipettes [1,2], microelectrodes [3], nanowire electrodes [4,5], and 3D nanoelectrodes [6], are extremely useful for 2D monolayered cells, but cannot access cells that are within a 3D construct. Numerous works were attempted to develop, engineer, and integrate novel biointerfaces that allow for intimate bioelectrical modulation within 3D cellular constructs and tissues. Such devices include nano-field effect transistors [[7], [8], [9]] and microelectrodes [[9], [10], [11]], which enabled 3D mapping and intra-volumetric electrical stimulation (ES) of engineered tissues. However, while these 3D bioelectronics offer many new capabilities, the stimulating electrodes are still limited to a scale of ∼20 μm, which enables single-cell but not subcellular resolution. Recently, a 3D transistor array was developed for single-cell intracellular recording within a 3D context [12]. However, the extremely sophisticated device fabrication and complexity of the biointerfaces limit their dissemination into standard 3D tissue culture. Moreover, nano-transistors are highly efficient for recording, but their electrical modulation capabilities are limited due to their high impedance. Optogenetics can achieve cellular activation with high spatial resolution; however, the need for genetic modification is considered its Achilles' heel, limiting its translational applications, especially in nonhuman primates and other clinically relevant models [13]. Other leadless and minimally invasive approaches for electrical modulation [14], which are based on magnetic [15,16] or ultrasonic [17,18] modulation, lack single-cell spatial resolution. Consequently, despite the exponential growth of bioelectronic research, the ‘toolkit’ available for such investigations fails to meet the level of complexity required in terms of spatial resolution and leadless capabilities.

Optoelectronic materials possess the potential to overcome such limitations as they enable leadless and minimally invasive optoelectronic modulation. For instance, organic photocapacitors [19], bioresorbable silicon p-n diodes [20], and porosity-based silicon heterojunction [21] thin-films were used to transduce optical illumination into electrical stimulation of nerve and heart tissues ex vivo and in vivo. For applications requiring high spatial resolution, optoelectronic silicon nanowires (SiNWs) have achieved local stimulation with subcellular resolution [14,[22], [23], [24], [25]]. The optoelectronic modulation is achieved using a core-shell configuration with dopant modulation. By using the chemical vapor deposition (CVD) process, the core is introduced with boron doping to form a p-type core, and then a shell with intrinsic silicon and a 2^nd^ shell with phosphorus doping to form an n-type outer shell. The resulting diode structure can serve as a photocathodic nanodevice that can generate local faradaic photoelectrochemical output [25]. Moreover, the ability of SiNWs to spontaneously internalize into many cell types [22,23,26] allows simple and minimally invasive intracellular access. Indeed, many studies have demonstrated local electrical modulation of 2D monolayered cells [14,[22], [23], [24], [25]] and ex vivo 3D cardiac tissues with SiNWs [23]. Moreover, SiNWs introduced into cardiac organoids improved the recovery of infarcted hearts [27], but no ES was attempted in this study.

When considering 3D engineered tissue constructs, the study of cellular interactions is critical for understanding tissue-level behaviors that cannot be replicated in traditional 2D monolayers [1]. While 2D systems have provided foundational insights into cell biology, they fail to fully capture the complex spatial and biophysical cues present in native tissues. For instance, although heterocellular electrical coupling between cardiomyocytes (CMs) and myofibroblasts (MFs) is prevalent in 2D monolayers [28,29], their prevalence in native cardiac tissue is debated [[30], [31], [32], [33]]. This is due to the aforementioned challenge of performing intracellular electrical modulation of a specific cell (MF) within a viable 3D tissue. Consequently, modeling the arrhythmogenic effect of MFs-rich scar tissue generated after myocardial infarction is dramatically limited by this open question. However, to date, there is no available platform that enables local intracellular electrical modulation of a specific cell within a 3D engineered tissue construct. Thus, a new tool that will allow leadless electrical modulation of cells inside a three-dimensional construct may open new ways for fundamental electrophysiological and bioelectronic research.

This study developed a novel 3D cellular construct, termed the **e-scaffold**, which integrates free-standing SiNWs within a biocompatible matrix. The scaffold enables the culture of cells in a 3D configuration, providing a more physiologically relevant context for studying intercellular interactions. Importantly, the embedded SiNWs can be internalized by various cell types, thereby forming intracellular biointerfaces that facilitate localized electrical modulation through point-laser illumination. The effect of impregnated SiNWs on scaffold geometry and dermal fibroblast viability was assessed. In addition, calcium imaging was performed to demonstrate that electrical modulation via SiNWs can induce intercellular calcium wave propagation, highlighting the potential of the e-scaffold to facilitate electrical communication between cells in 3D. Building on this proof-of-concept, we investigated the electrical coupling of cardiac myocytes and MFs within the e-scaffold, in attempt to tackle the long-standing debate regarding in vivo heterocellular coupling. The findings suggest that the 3D context has a significant influence on the extent and nature of cellular coupling compared to the 2D context, underscoring the importance of using physiologically relevant models for studying tissue-level phenomena. Moreover, they highlight the utility and validity of the e-scaffold for maintaining a viable 3D tissue construct and performing 3D modulation with subcellular precision. This work not only advances our understanding of cellular electrical coupling of cardiac cells in 3D but also establishes a versatile tool for broader applications in tissue engineering and regenerative medicine. It offers precise spatial and temporal control over cellular stimulation, creating new avenues for studying cell signaling and intercellular coupling in a comprehensive 3D cellular construct. The integration of optoelectronic SiNWs into 3D scaffolds paves the way for innovative approaches to study and modulate cellular behavior in complex tissue systems.

## 2 Results and discussion

**Fig. 1:**
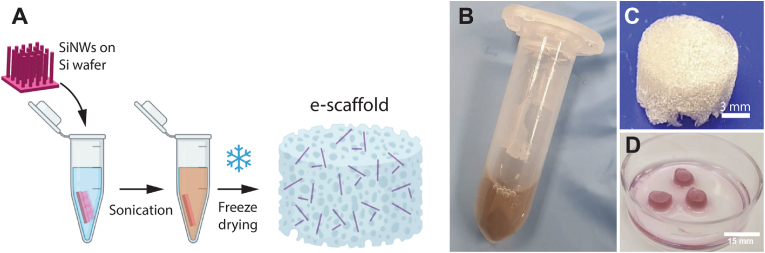
E-scaffold fabrication: (A) the e-scaffold fabrication workflow includes sonication of the silicon nanowires (SiNWs) in the gel, crosslinking and freeze-drying. (B) The resulting alginate gel with sonicated free-standing SiNWs, and the fabricated e-scaffold after (C) freeze-drying, and (D) immersion in medium. (For interpretation of the references to color in this figure legend, the reader is referred to the Web version of this article.)

**Fig. 2:**
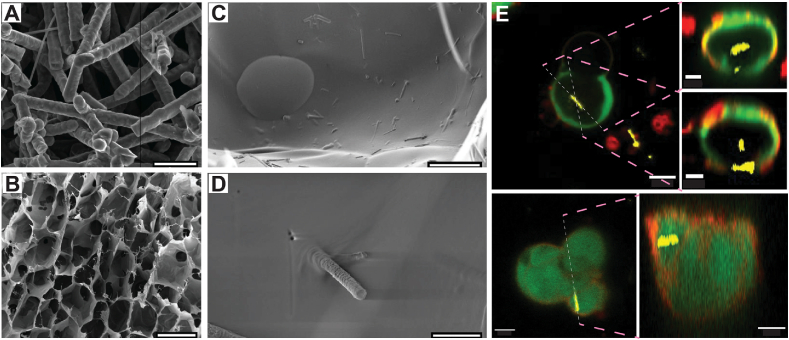
The e-scaffold: Scanning electron microscopy (SEM) images of (A) silicon nanowires (SiNWs) before sonication (scale bar is 2 μm), and (B) of the e-scaffold with impregnated SiNWs (scale bar is 200 μm). (C–D) High-magnification SEM images of the SiNWs (C) lying on the alginate scaffold (scale bar is 20 μm) or (D) protruding out of its surface (scale bar is 1.5 μm). (E) Left panels: confocal microscopy images of NHDF cells (red-membrane, green-cytosol) and internalized SiNWs (reflected light, yellow). Each image represents a single plane from a z-stack (scale bar is 10 μm). Right panels: a cross-sectional view of the lines highlighted in the left panel that verifies the SINWs' intracellular location (scale bars of top two inserts are 5 μm, and 10 μm for bottom insert). (For interpretation of the references to color in this figure legend, the reader is referred to the Web version of this article.)

In this study, a 3D scaffold with impregnated SiNWs was developed for optical intracellular activation and electrical coupling of cultured cells. As a simplified 3D scaffold, we used a modified protocol involving alginate hydrogel and the freeze-drying technique [[34], [35], [36]]. Free-standing SiNWs were suspended in the alginate solution before crosslinking, by sonicating a silicon wafer chip with p-i-n SiNWs grown on it via CVD (Fig. 1A). After sonication, we obtained a suspension of SiNWs and used SEM imaging to visualize the SiNWs; their diameter was 355.9nm±47.4nm(STDV) and their length 5.2μm±3.5μm(STDV). The resulting brownish SiNWs-alginate solution (Fig. 1B) was then ionically crosslinked and freeze-dried to form a solid white scaffold with a brownish appearance (Fig. 1C). The scaffolds maintained their 3D geometry when hydrated with culture media, without signs of disintegration (Fig. 1D). Fig. 2A shows a scanning electron microscopy (SEM) image of the SiNWs used for e-scaffold fabrication before sonication. We used SEM imaging to visualize and assess the prevalence of SiNWs at the surface of the scaffold's pores. Herein, we aimed for traceable amounts of SiNWs that may be visualized at the open area of the pores located at the scaffold surface. As SiNWs were evenly dispersed within the gel prior to freezing, we assume that they are consequently evenly dispersed within the scaffold. Under this assumption, we believe that the visualized top pores are a fair representation of the internal pores within the scaffold. A closer examination of the scaffold reveals that most of the SiNWs were lying on or within the wall of the scaffold's pores. However, several SiNWs were randomly oriented and protruded from the alginate wall (Fig. 2D). As these SiNWs are intended to form intracellular biointerfaces, we then investigated whether they are free to be internalized by the interfacing cells by seeding Normal human dermal fibroblasts (NHDFs) on the scaffold. Indeed, Fig. 2E shows that NHDF cells were able to uptake the SiNWs within the e-scaffold. Confocal imaging of NHDFs reveals that the SiNWs are internalized within the cells, as corroborated by a cross-sectional view of the 3D z-stacks. The underlying mechanism for SiNWs uptake may be attributed to phagocytosis, as was shown for SiNWs in 2D monolayers [26]. However, although cells in a 3D microenvironment may follow different uptake mechanisms than their 2D counterparts, this study only verified the cellular uptake of SiNWs, while the underlying mechanism is outside the scope of this study. Yet, the cells were rounded and did not form intercellular networks and interactions. This may be attributed to the use of alginate, an inert polymer that does not support cell-extracellular matrix (ECM) interactions [37]. In conclusion, alginate-based e-scaffolds enable cells to internalize SiNWs and form an intracellular biointerface; however, they are insufficient to support a viable and functional engineered tissue.

**Fig. 3:**
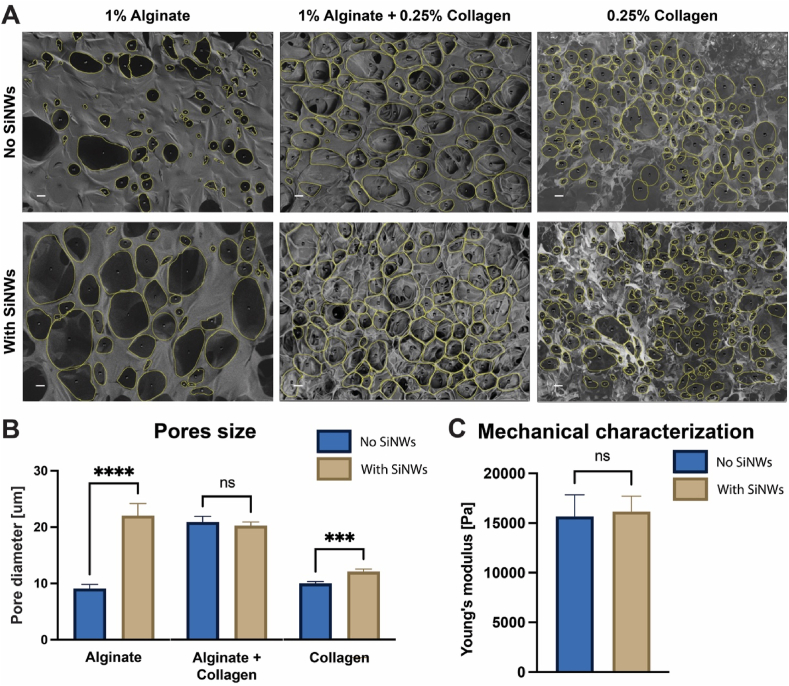
SiNWs effect on scaffold macroporous structures: (A) Scanning electron microscopy (SEM) images of scaffolds comprised of alginate (left), alginate and collagen (middle) or collagen (right), without (top) or with (bottom) impregnated silicon nanowires (SiNWs). Scale bars are 20 μm. (B) Effect of SiNWs on the pore size in each scaffold type. T-test ∗∗∗p < 0.001 and ∗∗∗∗p < 0.0001. n = 3 scaffolds in each group, with five randomly selected images from each scaffold. (C) Effect of SiNWs on the mechanical properties of VLVG alginate scaffolds. Young's modulus was calculated from rheology measurements. T-test p = 0.86. n = 3–4 scaffolds in each group. (For interpretation of the references to color in this figure legend, the reader is referred to the Web version of this article.)

To address this issue, two approaches were taken to generate scaffolds that promote cell-ECM interactions: the incorporation of collagen into the scaffolds or the use of RGD peptide-decorated alginate. To analyze these scaffolds, we used SEM imaging to visualize the scaffolds and verify the presence and availability of SiNWs within them, as well as to analyze their macropore structure. Fig. 3A shows SEM images of scaffolds made of alginate, collagen, and alginate/collagen hybrids. To assess the effect of SiNWs on scaffold porosity, we fabricated scaffolds with or without SiNWs out of each formulation. The addition of SiNWs had varying effects, depending on the type of scaffold, with a tendency to increase the size of the resulting pores (Fig. 3B). While alginate scaffolds with SiNWs yielded pores larger by a factor of ∼2.4, collagen scaffolds with SiNWs had ∼1.2-fold larger pores than their neat counterparts. A combination of alginate and collagen had no apparent effect on pore sizes. This may be attributed to several phenomena that can affect the freeze-drying process. For instance, ice nucleation, viscosity reduction, and polymer self-assembly may all be affected by the presence of SiNWs, which may contribute differently to the nature of pore formation. All pore structures obtained using the different alginate/collagen concentrations, with or without SiNWs, were suitable for 3D tissue culture and, therefore, were not further optimized in this work. However, if a specific pore size is required for a specific tissue engineering application, the polymer/SiNWs concentrations and ratio may be optimized to obtain the desired structure. Moreover, Fig. 3C shows that the presence of SiNWs does not affect the mechanical properties of the e-scaffolds as demonstrated by the comparable Young's modulus.

**Fig. 4:**
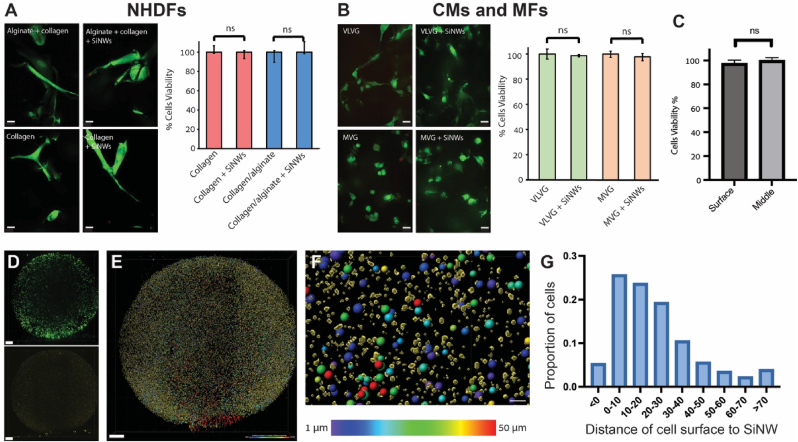
Live/dead assay: (A) Confocal microscopy images of normal human dermal fibroblasts (NHDFs) seeded on e-scaffolds comprised of alginate and/or collagen, with or without embedded silicon nanowires (SiNWs) (left). Cells were labeled with Calcein (green, live) and propidium iodide (red, dead). Scale bars are 20 μm. Right panel: Effect of SiNWs on the viability of the NHDFs. (B) Confocal microscopy images of primary cardiomyocytes (CMs) and myofibroblasts (MFs) seeded on e-scaffolds comprised of VLVG or MVG alginate, with or without SiNWs (left). Cells were labeled with Calcein (green, live) and propidium iodide (red, dead). Scale bars are 20 μm. Right panel: Effect of SiNWs on the viability of the CMs and MFs. (C) Comparing the viability of cells at the surface and the inner part of the cells. n = 3 scaffolds in each group, with 10 randomly selected images from each scaffold. (D) Light sheet microscopy imaging of RGD-decorated alginate e-scaffold (MVG) seeded with cardiac cells. Live cells were stained with Calcein (green, top image), and SiNWs were visualized using reflection mode (yellow, bottom image). Scale bars are 500 μm (E) The 3D reconstruction in D was analyzed using Imaris software, and spots and surfaces were generated to represent the cells (colored spheres) and SiNWs (yellow surfaces). The cells were color-coded by the shortest distance to a SiNW. Scale bar is 500 μm (F) An enlarged view of the cells and SiNWs from E. Scale bar is 50 μm (G) Distribution of cells according to the distance to the closest SiNW. Negative values indicate SiNWs that are within a cell (see more data in Supplementary Fig. S1). (For interpretation of the references to color in this figure legend, the reader is referred to the Web version of this article.)

To demonstrate the utility of the fabricated e-scaffolds to culture cells in a 3D context, we used collagen to enhance cell-ECM interaction or collagen/alginate hybrids. The hybrids were designed so that the alginate provided stable mechanical support, while collagen enhanced cellular interactions. Indeed, confocal imaging of NHDF cells after 7 days in 3D culture show that cells were viable, and that the impregnated SiNWs had no apparent effect on cell viability or morphology (Fig. 4A). However, although these scaffolds supported 3D cellular integration, their overall dimensions were not stable, and the scaffolds shrank. Therefore, for applications that require scaffold integrity at the macro scale and controlled degradability (in addition to no immunogenicity), pure alginate scaffolds (collagen-free) may be advantageous [38,39]. To this end, an e-scaffold of RGD-decorated alginate with impregnated SiNWs was developed. The RGD decoration facilitated cell-alginate interactions, allowing cells to integrate within the e-scaffold while overcoming the limitations of collagen with respect to mechanical stability and biodegradability. Fig. 4B shows cardiac cells, CMs and MFs, grown within MVG and VLVG alginate scaffolds. Both types of scaffolds demonstrated their utility for 3D cardiac cell culture. Cellular viability assays showed that the presence of impregnated SiNWs did not affect the cells. Moreover, when comparing the viability of the cells at the inner part of the e-scaffolds (∼ 1 mm deep) to its surface, no difference was observed (Fi. 4C). This can be attributed to the fact that cellular accumulation at the surface was due to cell seeding and low cellular penetration into the e-scaffolds, and not due to cell death due to lack of nutrients. Fig. 4 D (and Supplementary Fig. S1) shows light sheet imaging of the full e-scaffold scan. These scans show that the overall dimensions of the e-scaffolds were maintained intact (the same ∼6 mm diameter made in a 96-well plate). Moreover, the SiNWs appear to be evenly distributed within the scaffold, whereas the cells tend to accumulate at the scaffold perimeter, a typical characteristic of 3D engineered constructs. We then used the Imaris software to identify and segment individual cells and to analyze the distance between the surface of each cell to the closest SiNWs. In this setting, negative distances represent cells that have SiNWs within their volume (Fig. 4E–G, and Supplementary Fig. S1). Using this analysis, we show that for MVG and VLVG decorated alginate e-scaffolds, the proportions of cells with internalized SiNWs were 5.3 % and 2.7 %, respectively. For applications in which a larger proportion of cells with internalized SiNWs are required, the concentration of SiNWs within the e-scaffolds may be increased. On the other hand, increasing SiNW concentration may also increase cellular invasiveness. Thus, since these proportions were sufficient for performing our electrical coupling investigation, we used the scaffolds as is for 3D intercellular investigations. Moreover, to verify that cells can withstand higher SiNWs concentrations, we performed an exaggerated exposure of NHDFs and cardiac cells to increasing SiNWs concentrations. However, for 2D monolayers all SiNWs are expected to eventually settle down on the cells, while in 3D they are fixed in the e-scaffold. Consequently, comparing SiNWs concentration in 2D and 3D is challenging, as cells in 3D are exposed to fewer SiNWs for a given concentration. Supplementary Fig. S2 shows the effect of increasing concentration. Note that while we used 2 mm^2^/ml (area of CVD grown chip/ml alginate), using 1.25 mm^2^/ml resulted in >80 % internalized with SiNWs, which increases to ∼ 100 % for higher concentrations without any apparent effect on cellular viability. For cardiac cells, a similar trend was observed, with a minor decrease in viability from 100 % to above 95.2 % for SiNWs concentration of 1.25 mm^2^/ml. These results show that SiNWs concentration may be increased dramatically without a major effect on cellular viability. We also used SEM imaging to visualize cells cultured on the scaffolds. NHDFs cultured on MVG alginate scaffolds show that cells on the surface of the scaffold did not show any apparent difference when cultured on SiNWs impregnated e-scaffolds (Supplementary Fig. S3). Overall, these results suggest that the impregnation of SiNWs within 3D scaffolds is a viable means of introducing an optoelectronic interface to cells in a 3D context. Although cellular density within the 3D scaffold is lower than physiological densities or 2D monolayers, the general e-scaffold approach can be combined with other tissue engineering approaches that facilitate enhanced cellular densities. Lastly, they demonstrate that the platform is highly versatile and compatible with various scaffold formulations, including alginate, collagen, RGD-peptide-decorated materials, and combinations thereof. Together, these findings suggest that the developed platform can be easily adopted and tailored for specific tissue engineering requirements.

**Fig. 5:**
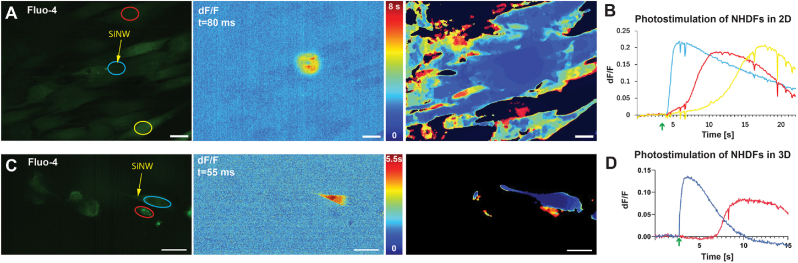
Optoelectronic modulation of normal human dermal fibroblasts in 2D and 3D: (A) Left: confocal microscopy image of (normal human dermal fibroblasts) NHDFs grown in 2D monolayers on a glass dish. Free standing silicon nanowire (SiNW) were seeded on the NHDFs, rinsed off, and NHDFs were loaded a calcium indicator (fluo-4). The location of the stimulated SiNW is marked with a yellow arrow and the different cells (regions of interest (ROIs)) that were analyzed are marked with circles. Middle: dF/F image 80 ms after the optical stimulation illustrates the local calcium flux originating at the stimulation site. Right: heat map of calcium flux propagation showing the temporal distribution of calcium netween the cells. Scale bars are 25 μm. (B) dF/F values over time of the ROIs from A. Optical stimulation time is marked with a green arrow (C) Left: confocal microscopy image of NHDFs grown in a 3D e-scaffold (with impregnated SiNWs) loaded with a calcium indicator (fluo-4). The location of the stimulated SiNW is marked with a yellow arrow and the different cells (ROIs) that were analyzed are marked with circles and arrows. Middle: dF/F image 55 ms after the optical stimulation illustrates the local calcium flux originating at the stimulation site. Right: heat map of calcium flux propagation showing the temporal distribution of calcium between the cells. Scale bars are 25 μm. (D) dF/F values over time of the ROIs from A. Optical stimulation time is marked with a green arrow. (For interpretation of the references to color in this figure legend, the reader is referred to the Web version of this article.)

After demonstrating that the e-scaffolds can serve as the foundation for viable 3D cellular constructs, their utility for subcellular bioelectric modulation of cultured cells was assessed using our previously reported protocol for investigating intercellular electrical coupling in 2D monolayers [23,40]. To assess the spatial resolution of the optoelectronic modulation, we consider both the size of the point illumination and the SiNWs as the limiting factors. Herein, the modulation was applied using a collimated laser beam that was focused to a diffraction-limited spot using the confocal objective. For a collimated 640 nm laser beam focused via a 1.1 NA objective, the spot size was $\frac{\lambda }{2NA}≅290\;nm$. Moreover, the size of the SiNWs is about 350 nm in diameter, and ∼ 5 μm long. Thus, since electrical stimulation is confined to the location where light and SiNWs coincide, sub-cellular resolution can be achieved as we have previously reported [22]. Upon optical intracellular modulation of an NHDF cell in a a 2D monolayer, a calcium wave originating at the location of the stimulated SiNW propagated within the cell (Fig. 5A–B, and supplementary video S1). To demonstrate the spatial resolution, dF/F image of the cells 80 ms after optical stimulation shows a local calcium flux before it diffuses throughout the cell. Although the size of the excited flux is indeed larger than the SiNW and the laser spot, this may be attributed to the temporal resolution of the optical imaging, which limits the ability to visualize the initial origin of the calcium flux. Then, the electrical effect was mitigated to neighboring cells via electrical coupling as evident by the gradual propagation of calcium from the stimulated cell to its neighbors (Fig. 5A–B and supplementary video S1). The photogenerated response induced by the SiNWs, may be attributed to the photoelectrochemical properties of the coaxial SiNWs with p-i-n diode structure [25]. Although several other mechanisms may contribute to the optoelectronic modulation, such as photo-generated reactive oxygen species (ROS), transient thermal poration, or thermally induced capacitive currents, we focused on exploiting the photogenerated response and not on studying the underlaying mechanism which is outside the scope of this study. We then used the same cells in a 3D e-scaffold to demonstrate its ability to support growth and electrical coupling in 3D. Indeed, optical stimulation of internalized SiNWs resulted in a calcium response which was detected by imaging the calcium indicator in 14 out 18 attempts (78 %). Although SiNWs appeared internalized in all stimulated cells, we assume that for non-responsive cells they were outside the cells or in-between two adjacent cells. This is because the actual location is difficult to assess during live imaging without proper plasma membrane staining. However, since we are investigating intercellular communication, it is reasonable to evaluate the response of neighboring cells only for successful optical stimulation, which can be defined as stimulations that triggered a reaction within the stimulated cell. Fig. 5C–D (and supplementary video S2) shows a representative response in which a Calcium flux across NHDF cells cultured in in 3D scaffolds followed a similar trend to the 2D modulation, but was somewhat delayed compared to the 2D monolayers. This may be due to radial calcium propagation to neighboring cells, whereas the capture of the phenomenon by confocal microscopy is limited to the x-y directions only. Since the propagation in 2D monolayers is confined to the x-y direction, and the fact that the larger the area, the slower the propagation is, the overall propagation was faster in 2D culture. Moreover, in some cases, the calcium propagated between cells that did not appear to be in direct contact. It is important to note that the imaging was performed using a spinning disc confocal microscope, which does not image cells that are above and below the focal plane. Yet, these cells may be responsible for the electrical coupling via out-of-plane routes. These results demonstrate the utility of the e-scaffold for 3D investigations of the bioelectrical coupling phenomenon in a straightforward and simple manner.

The e-scaffold was then applied to address the longstanding debate regarding the existence of heterocellular electrical coupling between MFs and CMs in 3D tissue constructs [[41], [42], [43]]. While such coupling is well documented in 2D monolayered cells [28,44,45], its in vivo prevalence is still debated. As 2D monolayered cells are a poor electrophysiological model of the 3D native tissue, we used the e-scaffold to study this intercellular electrical communication in 3D cardiac cellular constructs. Although optogenetics offers ways to electrically modulate a specific cell within a 3D context [[46], [47], [48]], it relies on the availability of transgenic animal models (generally mice). However, from an electrophysiological perspective, mice are considered of poor human relevance as compared to other animal models [49,50]. On the other hand, available small-animal models, such as rabbits and guinea pigs, do not have available transgenic animal strains that will allow optogenetics. Thus, it is essential to develop a nongenetic approach for electrically modulating cells within 3D construct with high spatial precision.

**Fig. 6:**
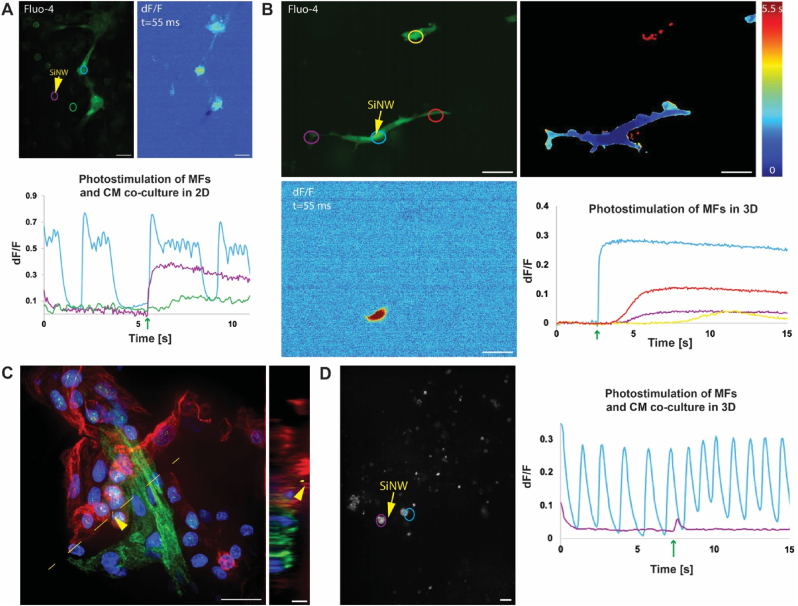
Optoelectronic modulation of cardiac cells in 2D and 3D: (A) Top left: confocal microscopy image of cardiomyocytes (CMs) and myofibroblasts (MFs) grown in 2D monolayers on a glass dish. Free standing silicon nanowire (SiNW) were seeded on the CMs-MFs co-culture, rinsed off, and cells were loaded with calcium indicator (fluo-4). The location of the stimulated SiNW is marked with a yellow arrow and the different cells (regions of interest (ROIs)) that were analyzed are marked with circles. Top right: dF/F image 55 ms after the optical stimulation illustrates the stimulated MFs and neighboring CMs with elevated calcium levels. Bottom: dF/F values over time of the highlighted ROIs from top left panel. Optical stimulation time is marked with a green arrow. Scale bars are 25 μm. (B) Top left: confocal microscopy image of MFs grown in a 3D e-scaffold loaded with a calcium indicator (fluo-4). The location of the stimulated SiNW is marked with a yellow arrow and the different cells (ROIs) that were analyzed are marked with circles and arrows. Top right: heat map of calcium flux propagation showing the temporal distribution of calcium within the cells. Bottom left: dF/F image 55 ms after the optical stimulation illustrates the local calcium flux originating at the stimulation site. Bottom right: dF/F values over time of the highlighted ROIs from top left panel. Optical stimulation time is marked with a green arrow. Scale bars are 25 μm. (C) Left: representative confocal microscopy image of immunohistochemically analyzed samples showing the SiNW (reflection, yellow) internalized into MFs (vimentin, red) and not CMs (cardiac troponin, green). Scale bar is 20 μm. Right: a cross-sectional view of the yellow line highlighted in the left image to verify SiNWs location within the MF. Internalized SiNWs are marked with yellow arrowhead. Scale bar is 5 μm. (D) Left: confocal microscopy image of CMs and MFs grown in a 3D e-scaffold loaded with a calcium indicator (fluo-4). The location of the stimulated SiNW is marked with a yellow arrow and the different cells (ROIs) that were analyzed are marked with circles. Scale bar is 25 μm. Right: dF/F values over time of the highlighted ROIs from left image. Optical stimulation time is marked with a green arrow. (For interpretation of the references to color in this figure legend, the reader is referred to the Web version of this article.)

To this end, we employed our e-scaffold to compare intercellular electrical coupling in 2D vs. 3D cultures. We first cultured primary isolated cardiac cells (CMs and MFs) in a 2D monolayer, seeded SiNWs on them, and allowed them to internalize. SiNWs have been shown to internalize spontaneously into MFs [22,23] but not into CMs [26], as validated here by visualizing the cells loaded with calcium indicator (fluo-4); cells with internalized SiNWs were non-excitable, whereas spontaneously beating CMs did not carry SiNWs. We identified MFs with internalized SiNWs, which were near CMs with low activity. Thereafter, upon optical stimulation of the MFs bearing SiNW, an immediate response of the neighboring CMs was observed, and manifested by firing of high-frequency action potentials (APs) (Fig. 6A and supplementary video S3). At a delay of up to ∼1.2 s, a subtle and slow calcium flux propagated inside the stimulated MFs (purple ROI) and to neighboring MFs (green ROI), which demonstrated MFs-MFs homocellular coupling in 2D. This electrical coupling relies on intercellular gap junctions (mainly connexin 43) that allow calcium to diffuse from one cell to another. To verify this mechanism, we used carbenoxolone to block intercellular coupling as previously demonstrated [23]. Indeed, intercellular coupling was blocked, and calcium flux was limited to the stimulated cells without any apparent effect on neighboring cells (Supplementary Fig. S4). We then used our e-scaffold to compare MFs-MFs and MFs-CMs coupling in 3D to that of 2D. We first investigated homocellular (MFs-MFs) coupling by culturing MFs in 3D e-scaffolds. Upon optical stimulation, a calcium response was observed in 13 out of 20 attempts (65 %), which was similar to the proportion of success observed for NHDFs (Supplementary Fig. S5). Fig. 6B (and supplementary video S4) demonstrates that MFs were able to couple, and calcium propagated from the stimulated cells to neighboring cells. As for NHDF cells, the calcium propagation was slower in the 3D construct than in the 2D monolayers, probably due to the same effect of calcium propagating in all three dimensions. In addition, calcium propagation was observed between cells that were seemingly not connected, probably via cells coupling the effect that were above or below the imaging plane. Lastly, we used a CMs-MFs co-culture to study heterocellular coupling in a 3D e-scaffold. As for the 2D monolayers, in this co-culture setting, both cell types (CMs and MFs) were interfaced with the impregnated SiNWs. We first validate our assumption that SiNWs internalize into MFs, and not into CMs, in 3D constructs, the same way they were previously reported to internalize in 2D monolayers [26]. HIC-labeled CMs (cardiac troponin, green) and MFs (vimentin, red) seamlessly integrated within the 3D e-scaffold, with SiNWs (reflection, yellow) taken up by MFs (yellow arrowhead) and not CMs (Fig. 6C). Moreover, imaging of the co-culture using a calcium indicator verified that stimulated SiNWs were located within cells that were not spontaneously active. As for the MFs culture, when optical stimulation was performed for the MFs-CMs coculture, a calcium response was observed in 7 out of 11 attempts (64 %, Supplementary Fig. S5). Local optical stimulation of the internalized SiNWs triggered a clear response in neighboring CMs (Fig. and supplementary video S5), typically characterized by an immediate AP, an increase in beating frequency and elevation in resting calcium levels. All three responses may be attributed to the diffusion of calcium from stimulated MFs to neighboring CMs, in which APs are governed by a calcium-induced calcium release mechanism [51]. Moreover, as calcium release from the MFs was slow (Fig. 5, Fig. 6), calcium flux continued after the termination of the AP, instigating the observed elevation of the resting calcium level and the faster AP firing rate. Fig. 6D shows an example in which the resting AP firing rate was about ∼0.74 ± 0.043 (SEM) Hz before the modulation and increased to ∼0.98 ± 0.036 (SEM) Hz, for a duration of ∼6 s, immediately after the modulation. An immediate increase in the resting calcium level accompanied this frequency increase. Moreover, the effect of the calcium flux diminished within a few seconds, as indicated by both the decrease in the resting calcium level and the corresponding decrease in frequency. On the other hand, the cellular MFs response was significantly different in the MFs-CMs co-culture when compared to that observed in the 2D co-culture and the 3D MFs culture (without CMs). Only a short calcium transient (∼500 ms), which immediately decayed and resumed its baseline level, was observed in stimulated MFs and was distinctly different from the long (>10 s) calcium fluxes that were observed in the 2D MFs-CMs co-culture and the 3D MFs culture. We postulate that the enhanced electrical coupling between MFs and CMs and the large volume of CMs in 3D, may have served as a calcium sink and buffered the calcium levels in MFs. Unlike MFs, CMs are adapted to handle abrupt changes in intracellular calcium, which are an integral part of APs. Consequently, Calcium from the MFs can diffuse to CMs via gap junctions and thereby be cleared by the CMs during normal AP calcium handling. Despite the prevalence of CMs-MFs coupling in 2D co-culture (Fig. 6A), the prolonged MFs response in 2D as compared to 3D co-cultures (Fig. 6D) may have been due to the 2D nature of the co-culture, in which CMs and MFs are only coupled at the edges of the spreading cells, while cells in 3D culture interact over a large cell surface area. Moreover, the flat morphology of the cells in 2D has a much smaller volume, which may further decrease the ionic capacity to serve as a calcium sink. Overall, these results clearly demonstrate the inherently different cell-cell interactions in 2D monolayers vs. 3D constructs. Although further research is essential to better characterize MFs-CMs interactions in 3D and then compare them to the in vivo microenvironment, the presented model still demonstrates the utility of the approach in studying intercellular electrical coupling in viable 3D contexts.

## 3 Conclusions

This e-scaffold presented here allowed for leadless and minimally invasive electrical modulation of cells within a viable 3D cellular construct. The e-scaffold was shown to allow cells to grow, interact, and couple. Cell viability was not affected by the presence of the free-standing SiNWs. To facilitate cell-ECM interaction, various polymers, including alginate and collagen, are suitable for fabricating microporous scaffolds that support cell-ECM interactions. Moreover, the SiNWs embedded within the e-scaffold were internalized by the interfacing cells and then exploited to induce local intracellular calcium transients optically. Intercellular calcium dynamics were used to assess the presence of electrical coupling between NHDF cells and cardiac cells. We demonstrated that cells in a 3D context, compared to a 2D context, exhibited enhanced electrical coupling. Overall, these results show that the e-scaffold may be a valuable, powerful, and most importantly, an easy and straightforward tool for bioelectronic modulation of 3D tissue constructs. The use of different cell types (cardiac cells and NHDF cell line) demonstrates the versatility and utility of our e-scaffold for various applications. Besides the SiNWs themselves, which can be fabricated using bottom-up [25,52] or top-down [53] approaches, e-scaffold fabrication was based on a standard freeze-drying technique, a method widely used in tissue engineering applications. The simplicity of this approach will enable the simple dissemination of versatile leadless electrical modulation of cells at extremely high spatial resolution.

## 4 Materials and methods

***SiNWs synthesis*:** Coaxial p-i-n-SiNWs were synthesized using an Au nanocluster-catalyzed chemical vapor deposition (CVD) process. Au colloidal nanoparticles (100-nm diameter; Ted Pella) were deposited onto Si substrates (Nova Electronic Materials) for use as catalysts. During the SiNW growth, silane (SiH_4_) was used as the Si reactant, diboron (B_2_H_6_, 100 ppm in H_2_) as the p-type dopant, phosphine (PH_3_, 1000 ppm in H_2_) as the n-type dopant, and hydrogen (H_2_) as the carrier gas. For the p-type core SiNW growth, SiH_4_, B_2_H_6_, and H_2_ were delivered at flow rates of 2, 10, and 60 standard cubic centimeters per min (sccm), respectively. P-type core SiNW growth was carried out for 30 min at 470 °C and 40 torr. For the intrinsic Si shell (i-shell) deposition, the temperature was ramped up to 650 °C, during which time no gas flow was allowed, and vacuum was applied. Then, SiH_4_ and H_2_ were delivered at 0.3 and 60 sccm, respectively, at 15 torr. For the n-type outer shell, PH_3_ gas was added at a flow rate of 1.5 sccm, under the same conditions. The shell depositions were performed at 750 °C at a pressure of 20 torr for 15 min per shell. Both intrinsic and n-type shells were grown for 20 min.

***E-scaffold fabrication*:** Scaffolds, with a diameter of 6 mm and a thickness of 2 mm, were prepared according to an adopted previously reported protocol [54,55]. We used alginate (LVG, NovaMatrix, Norway), alginate with RGD peptide decoration (VLVG or MVG, NovaMatrix, Norway), or collagen (Collagen 1, Rat Tail, Gibco). In brief, the alginates were dissolved in double-distilled water (DDW) to obtain a 1.2 % (w/v) solution. Then, a Si wafer with CVD-grown SiNWs was inserted into the solution (∼ 2 mm^2^ for 1 ml) and sonicated to suspend the free-standing SiNWs in the alginate solution. For the control scaffold without SiNWs, the last step was skipped. Then, the solution was cross-linked by adding calcium D-gluconate monohydrate (Sigma-Aldrich, 1.2 % (w/v)), under vortex to form a gel. The cross-linked alginate solution was poured into 96-well plates (100 μL/well), chilled to 2–8 °C overnight, frozen at −20 °C for 24 h, and lyophilized. Scaffold sterilization was achieved by exposure to UV light.

For collagen scaffolds, the SiNWs were sonicated into the solution of DDW, phosphate buffered saline x10 (PBS) (Sigma-Aldrich), and collagen (final concentration 2.5 mg/ml, Gibco). Only then the pH adjusted using NaOH 1N (Bio-Lab Ltd.) according to the manufacturer's instructions while the solution was cooled on ice. Thereafter, the solution was allowed to gel in a 37 °C incubator for 30 min, chilled to 2–8 °C overnight, frozen at −20 °C for 24 h, and lyophilized. Scaffold sterilization was achieved by exposure to UV light.

For alginate/collagen hybrids, we sonicated the SiNWs in a solution containing alginate and collagen and then the solution was titrated to pH = 7.2. Then, calcium D-gluconate monohydrate (Sigma-Aldrich, 1.2 % (w/v)) was added so that the final concentration will be 1 % alginate (w/v), 2.5 mg/ml collagen, and 0.2 % calcium D-gluconate monohydrate (w/v). Thereafter, the solution was allowed to gel in a 37 °C incubator for 30 min, chilled to 2–8 °C overnight, frozen at −20 °C for 24 h, and lyophilized. Scaffold sterilization was achieved by exposure to UV light.

For all types of scaffolds, control scaffolds without SiNWs were fabricated in the same way without the addition of SiNWs chips and sonication.

*Imaging of scaffolds pore structure*: Fabricated scaffolds were mounted on a SEM stub with conducting type and visualized in the confocal microscope. To assess pore's diameter, we used an average size as the pore were not rounded. To this end, we measured the area of visualized pores using ImageJ and derived the corresponding diameter.

*Mechanical characterization of alginate scaffolds*: Alginate scaffolds, with and without nanowires, were biomechanically characterized using an ARES G2 rheometer (TA instrument, New Castle, DE, USA) equipped with 20 mm hatch parallel plate geometry. Scaffolds were placed on the rheometer stage and compressed to 10 % strain. Strain (ε) and stress (σ) were calculated according to the following equations: $\varepsilon =\frac{l_{0}-l}{l_{0}}$ and $\sigma =\frac{N}{A}$, where $l_{0}$ and l are the initial and current lengths of the scaffolds, respectively, N is the applied axial force, and A is the surface area of the scaffold. Stress-strain curves were plotted (Supplementary Fig. S6), and Young's modulus was calculated as the slope of the linear fit.

***Cell culture*:** NHDF cell-line was kindly provided from the Stem Cell and Tissue Engineering Laboratory (prof. Shulamit Levenberg's lab) at the biomedical engineering faculty in Technion. Cells were cultured in Dulbecco's Modified Eagle's Medium - high glucose (DMEM) (Sigma-Aldrich) supplemented with 10 % (v/v) heat-inactivated fetal bovine serum (FBS, Gibco), 1 % (v/v) 2-Mercaptoethanol 50 mM (Gibco, 1 % (v/v) Penicillin-Streptomycin (PEN/STREP) (Sigma-Aldrich), and 0.2 %vol non-essential amino acids 100x (MEM-NEAA) (Biological industries).

Neonatal rat cardiomyocytes and myofibroblasts are isolated from rats up to 5 days old. The heart is carefully extracted and placed in a 10 cm dish containing ice-cold magnesium and calcium-free Hanks' Balanced Salt Solution (HBSS) from Biological Industries. After removing vessels and connective tissues from the ventricles, the heart is cut into small pieces and rinsed with ice-cold HBSS. These small heart pieces are then immersed in a 15 ml tube containing an enzyme mix obtained from the mouse-and-rat neonatal heart dissociation kit (Miltenyi Biotec). They are left to incubate for 15 min. After this incubation period, the tube contents are vigorously mixed by pipetting and then incubated for another 15 min before another mixing step. Once the components in the tube appear homogeneous, the cell suspension is filtered through a 70-μm strainer and combined with DMEM from Sigma-Aldrich, pre-warmed to 37 °C. This DMEM is supplemented with 10 % (v/v) FBS from Gibco, 1 % PEN/STREP, and 1 % L-Glutamine 200 mM from Gibco.

For 2D monolayers, both cell types, cardiac cells and NHDFs, $2.5\cdot 10^{5}$ cells were seeded onto glass bottom dishes coated with either collagen (NHDFs, 1 mg/ml rat type 1, Gibco) or fibronectin (cardiac cells, 1 mg/ml, Biological Industries). Cells were cultured for up to 7 days before performing optoelectronic modulation experiments. For 3D culture, $2\cdot 10^{6}$ cells were seeded onto a single scaffold (regardless the scaffold type), and cells were cultured for 7–10 days before performing optoelectronic modulation experiments.

***Cell staining*:** For 3D morphology and intracellular SiNWs visualization we used Calcein AM (abcam) viability staining and cytopainter (abcam) for cell plasma membrane staining. After 40 min incubation period, cells/scaffolds were subjected to three 5-min washes with their respective medium. For calcium imaging in photo-stimulation experiments we used calcium-sensitive dye (Fluo-4, abcam), following the same staining protocol. As SiNWs are not florescent, we used reflected light to image them using the confocal setting. For live-dead assay, both cell types were incubated in a humidified atmosphere at 37 °C, and the scaffolds were evaluated 7 days post-seeding. Cell viability within the 3D scaffolds was assessed using a non-destructive live/dead staining assay. Samples were washed with phosphate-buffered saline (PBS) and incubated for 30 min at 37 °C in a staining solution containing Calcein AM (0.004 mg/mL in HBSS) and propidium iodide (prepared by dissolving 1 mg in 6 mL PBS). Fluorescence microscopy was performed using a spinning disc confocal microscope, and image analysis was conducted with ImageJ software. Live (green cells) and dead (red nuclei) cells were counted, and the proportion of live cells was normalized to the control (no SiNWs) average. For comparing the surface to the inner part of the e-scaffolds, we scanned ∼ 100 μm of the surface and compared it to ∼ 1 mm inside the e-scaffolds. All experiments were performed in triplicate, and statistical analysis was conducted using two-way ANOVA.

*Light sheet Imaging and analysis*: The imaging was performed using the Light sheet 7 microscope with a Plan-Neofluar 5X lens. Cells were stained with Calcein and SiNW were visualized by their reflection. The e-scaffold was immersed in a 1 % agarose gel and then placed into a microscope holder using a syringe. Then, images were processed using the IMARIS software, which enabled a 3D reconstruction from the dataset. Each fluorescence channel was processed separately. For the Calcein imaging, we auto-created spots representing the cells, using a region-growing algorithm. The threshold was set for the cells as a surface area threshold of ≥915 μm^2^. The average cell diameter was defined as 25 μm, and a quality filter was applied to include only objects with quality >400. This quality value reflects a combination of fluorescence intensity and the object's proximity to the defined average diameter. For the SiNWs channel, surfaces were automatically generated to represent silicon nanowires using local contrast background subtraction and a region growing algorithm. The maximum diameter threshold was set to 8 [μm], with a seed point diameter of 4.5 [μm]. To reduce noise, voxels smaller than 10 were excluded. After defining spots (cells) and surfaces (SiNWs), the shortest distance from each spot (cell) to the nearest surface (SiNW) was computed using the Imaris "Shortest Distance" function. Each cell was color-coded based on this distance, with a scale bar ranging from 0 μm (purple) to 50 μm (red).

*SEM imaging*: To assess cell morphology on the scaffolds, NHDFs were seeded onto the samples and cultured for three days. Samples were rinsed with HEPES and fixed in 2 % Glutaraldehyde, 2 % Paraformaldehyde and 10 mM CaCl_2_ in 0.1 M Cacodylate buffer pH 7.4. Then the samples were post-fixed using 1 % Osmium Tetra-Oxide, dehydrated in graded ethanol series, critical point dried (Quorum K850) and coated with a layer of ∼3 nm of Iridium (Q150T Coater). Samples were then viewed on a Zeiss Ultra Plus HR Scanning Electron Microscope using a Secondary Electron detector.

*Optical stimulation of cell-nanowire composites*: Cells are seeded onto nanowire-embedded scaffolds and allowed to incubate for a period ranging from 7 to 10 d. After this incubation period, the cells are stained with Fluo-4 calcium dye (5uM) for 40 min and then thoroughly washed before being transferred to the spinning disc confocal microscope. Fluo-4 is excited using a 488 nm laser while SiNWs can be observed either under brightfield or by examining the reflected light. To induce specific SiNWs stimulation, a 640 nm laser pulse (10 ms, 10 mW, and 290–350 nm spot size) is employed (Supplementary Fig. S7). To analyze the data, we used the same previously reported data acquisition and analysis protocol we previously reported [56]. Changes in calcium transients are reflected in alterations in Fluo-4 intensity. Using ImageJ, a Δ(Fluorescence)/fluorescence (dF/F) for each pixel is generated, enabling us to analyze the signal effectively. heatmaps were generated by transforming the dF/F videos to a binary video and then color coded according to the propagation of the flux as previously reported [56] (Supplementary Fig. S8).

## CRediT authorship contribution statement

**Nadi Hathot:** Writing – original draft, Methodology, Investigation, Formal analysis, Data curation. **Tania Assaf:** Formal analysis, Data curation. **Layan Habib:** Formal analysis, Data curation. **Noa M. Cohen:** Methodology, Data curation. **Dana Nir:** Methodology, Data curation. **Alexander Borodetsky:** Methodology, Formal analysis, Data curation. **Shiri Karni-Ashkenazi:** Resources, Project administration, Data curation. **Menahem Y. Rotenberg:** Writing – original draft, Supervision, Resources, Project administration, Investigation, Funding acquisition, Formal analysis, Data curation, Conceptualization.

## Declaration of competing interest

The authors declare the following financial interests/personal relationships which may be considered as potential competing interests:Nadi Hathot, Tania Assaf, Layan Habib, Noa Cohen, Dana Nir, Alexander Borodetsky reports financial support, article publishing charges, equipment, drugs, or supplies, and writing assistance were provided by 10.13039/501100003977Israel Science Foundation (10.13039/501100003977ISF). Meanhem Y. Rotenberg has patent #WO2021076981 - METHODS AND SYSTEMS FOR MODULATING CELLULAR ACTIVATION issued to THE UNIVERSITY OF CHICAGO [US]/[US]. If there are other authors, they declare that they have no known competing financial interests or personal relationships that could have appeared to influence the work reported in this paper.

## Data Availability

Data will be made available on request.
