# Supplementary material for: Three-dimensional cellular construct with impregnated silicon nanowires for intracellular optoelectronic biointerface
Source: Mater Today Bio. 2025 Jul 2;33:102039. doi: 10.1016/j.mtbio.2025.102039 (PMC12273512; doi:10.1016/j.mtbio.2025.102039)
Supplement: Multimedia component 1 [file mmc1.pdf]

Supplementary information

Three-dimensional cellular construct with impregnated silicon nanowires for intracellular optoelectronic biointerface

Nadi Hathot[a,1], Tania Assaf[a,1], Layan Habib[a], Noa Cohen[a], Dana Nir[a], Alexander Borodetsky[a], Shiri Karni-Ashkenazi[a], and Menahem Y. Rotenberg[a,b,*]

[a] Department of Biomedical Engineering, Technion- Israel Institute of Technology, Haifa, 32000, Israel

[b] Russell Berrie Nanotechnology Institute, Technion - Israel Institute of Technology, Haifa, 32000, Israel

[1] These authors contributed equally to this work

* Corresponding author: hemir@technion.ac.il;

Supplementary information:

Figures S1-S8

Video S1- Calcium imaging and dF/F derived videos of the stimulation corresponding to figure 5A-B.

Video S2- Calcium imaging and dF/F derived videos of the stimulation corresponding to figure 5C-D.

Video S3- Calcium imaging and dF/F derived videos of the stimulation corresponding to figure 6A.

Video S4- Calcium imaging and dF/F derived videos of the stimulation corresponding to figure 6B.

Video S1- Calcium imaging and dF/F derived videos of the stimulation corresponding to figure 6D.

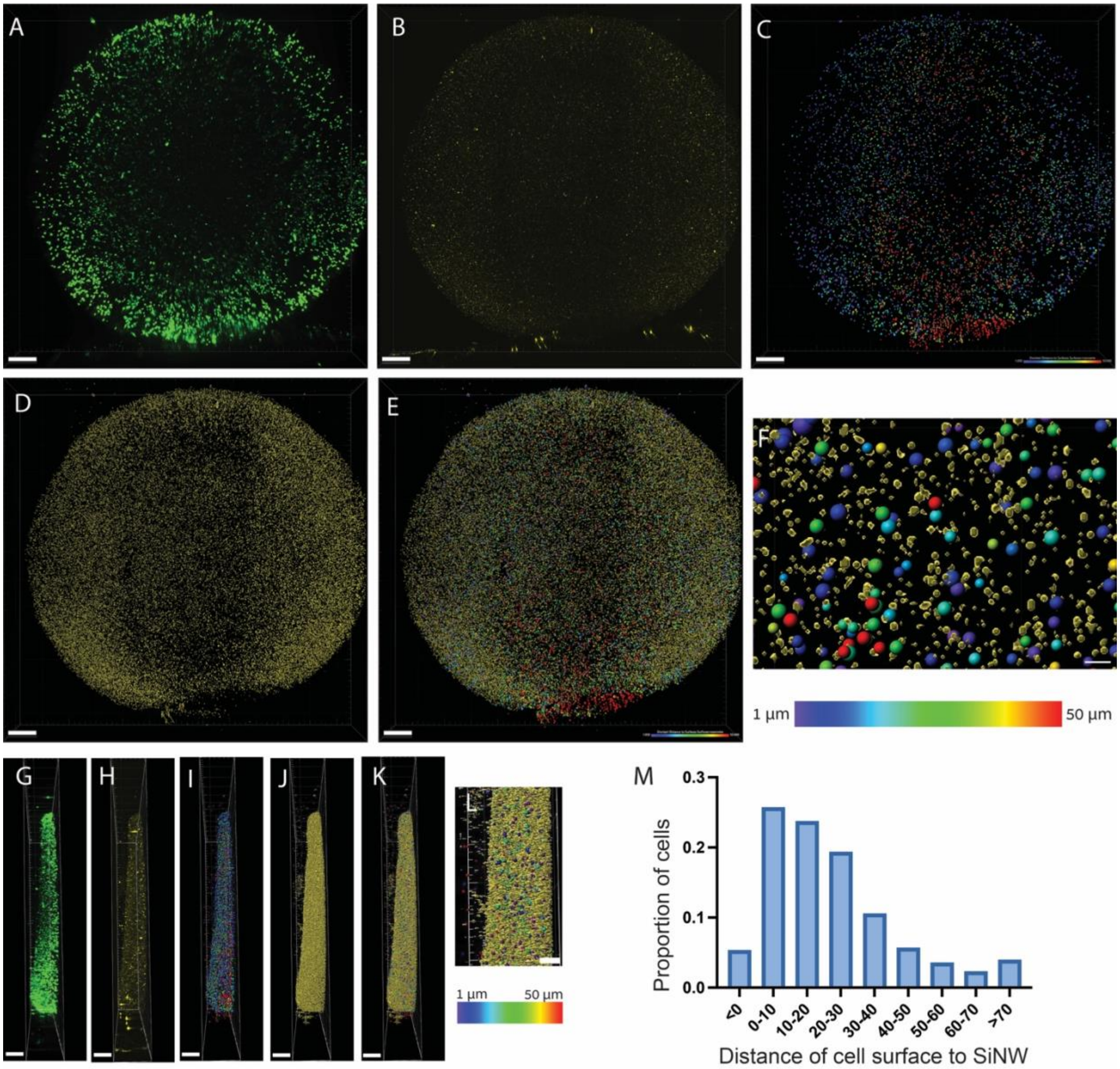

Figure S1: Cells and SiNWs distribution within the scaffolds. Light sheet microscopy imaging of RGD-decorated alginate e-scaffold (MVG) seeded with cardiac cells. (A) Live cells were stained with Calcein (green), and (B) SiNWs were visualized using reflection mode. Then, the 3D reconstruction in A and B was analyzed using Imaris software, and spots and surfaces were generated to represent the cells (C) and SiNWs (D), while the cells were color-coded by the shortest distance to a SiNW. (E-F) Frontal view of a merged image of C and D (E) and an enlarged view of the cells and SiNWs (F). (G-L) The side view of the same scaffold was generated to show the cross-sectional view of the e-scaffold. The same analysis was performed and presented in the same way as shown in A-F. Scale bars are 500 µm in A-E and G-K, 50 µm in F, and 200 µm in L. (M) Distribution of cells according to the distance to the closest SiNW. Negative values indicate SiNWs that are within a cell.

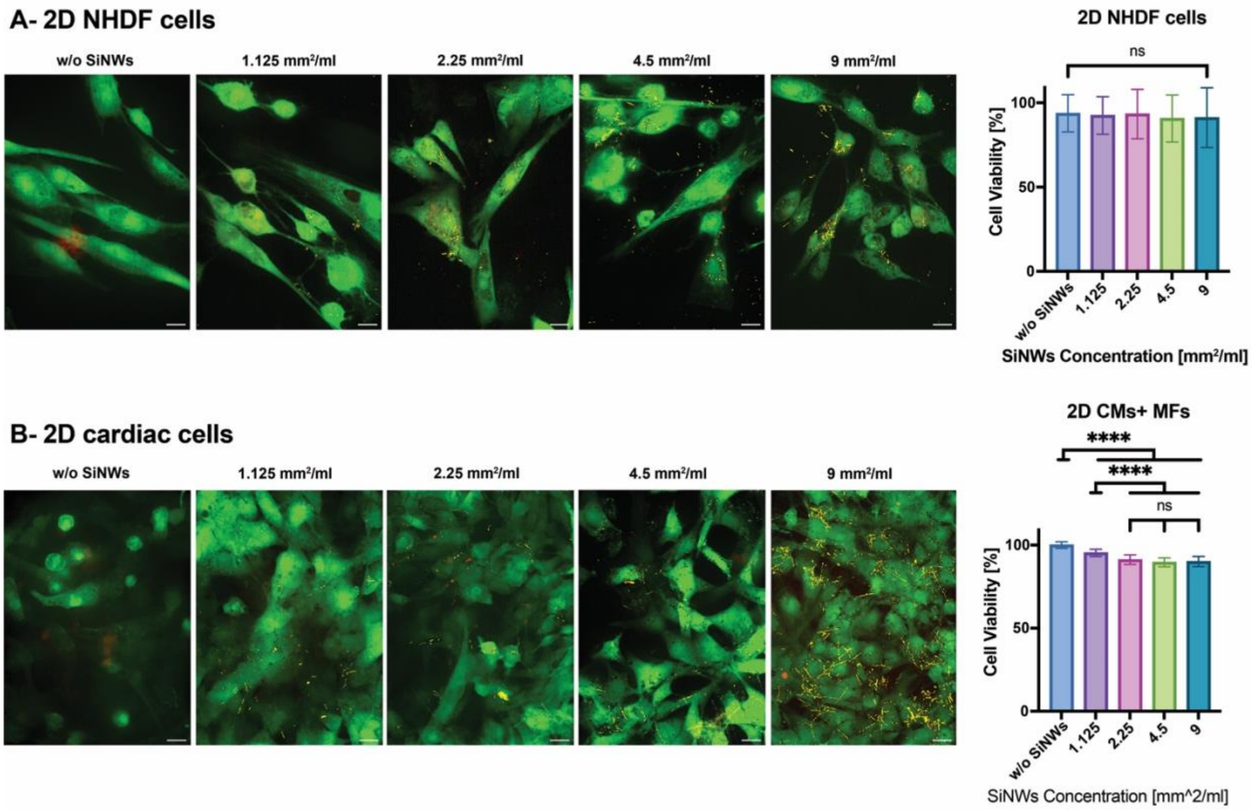

Figure S2: SiNW Toxicity. NHDFs (A) and cardiac cells (B) were labeled with Calcein (green, live) and propidium iodide (red, dead). Scale bars are 20 µm. The effect of SiNWs concentration on cellular viability is illustrated in the graphs on the right. ****- P<0.0001

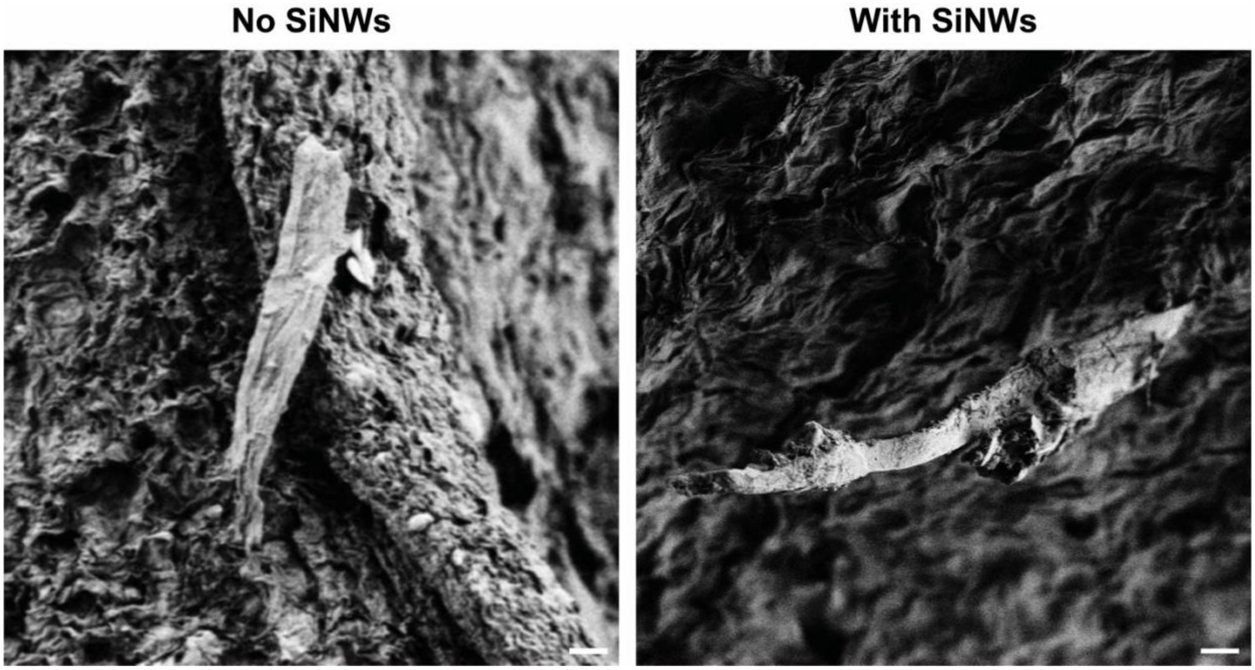

Figure S3: SEM images of NHDFs cultured on RGD-decorated alginate (MVG) scaffolds without (left) and with (right) SiNWs for 3 days. Scale bars are 10 μm.

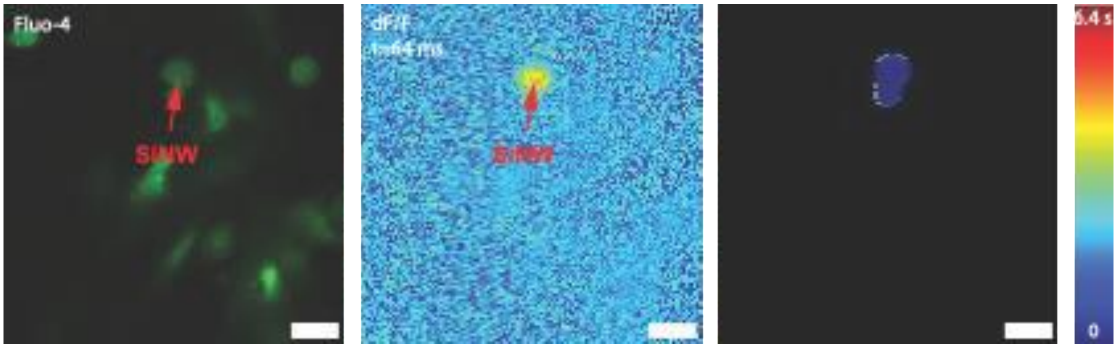

Figure S4: The role of connexin 43 in mediating intercellular electrical coupling. Representative example of the effect of carbenoxolone (connexin 43 blocker) on intercellular calcium propagation. Blocking connexin 43 gap junction completely abolished calcium propagation upon photo-stimulation. Left: confocal microscopy image of CMs and MFs co-culture in 2D loaded with a calcium indicator (fluo-4). The location of the stimulated SiNW is marked with a red arrow. Middle: dF/F image 64 ms after the optical stimulation illustrates the stimulated MF with elevated calcium levels. Right: heat map of calcium flux propagation showing that calcium flux was limited to the stimulated cell. Scale bars are 25 µm.

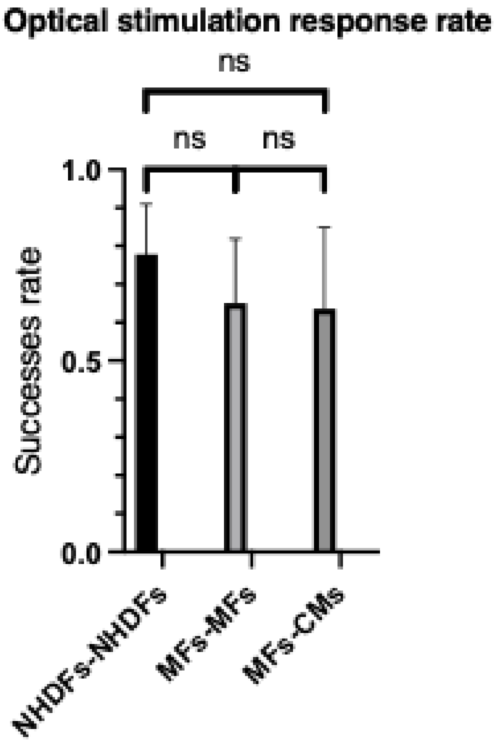

Figure S5: Optical stimulation success rate: The overall success rate of optical stimulation was determined by an apparent calcium response upon optical stimulation of a SiNW. Statistical significance was determined by calculating the 95% confidence intervals of the proportions using Wilson/Brown method.

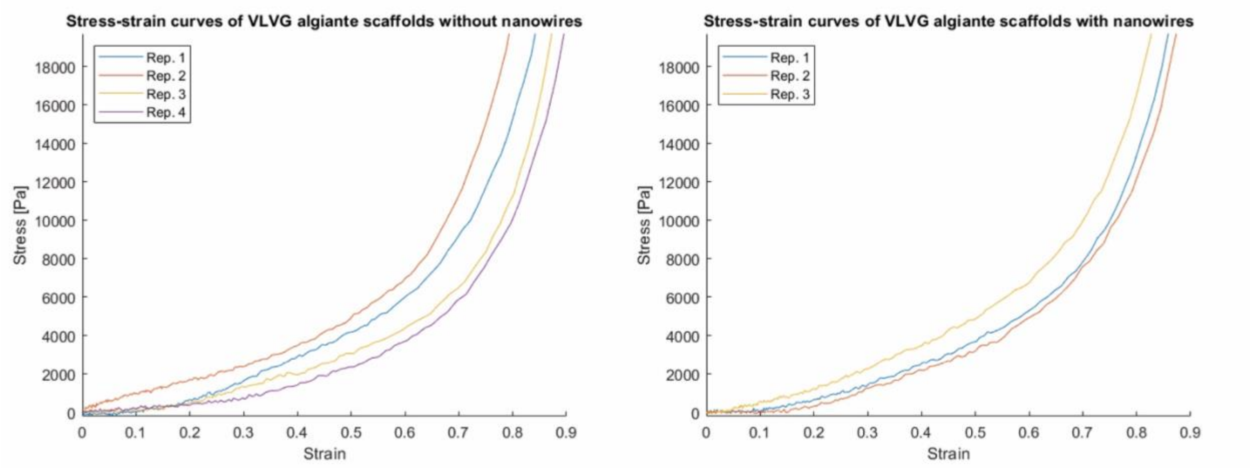

Figure S6: Stress-strain curves of VLVG alginate scaffolds without SiNWs (left) and with SiNWs (right). These curves were used to drive the scaffold's Young's modulus in Fig. 3.

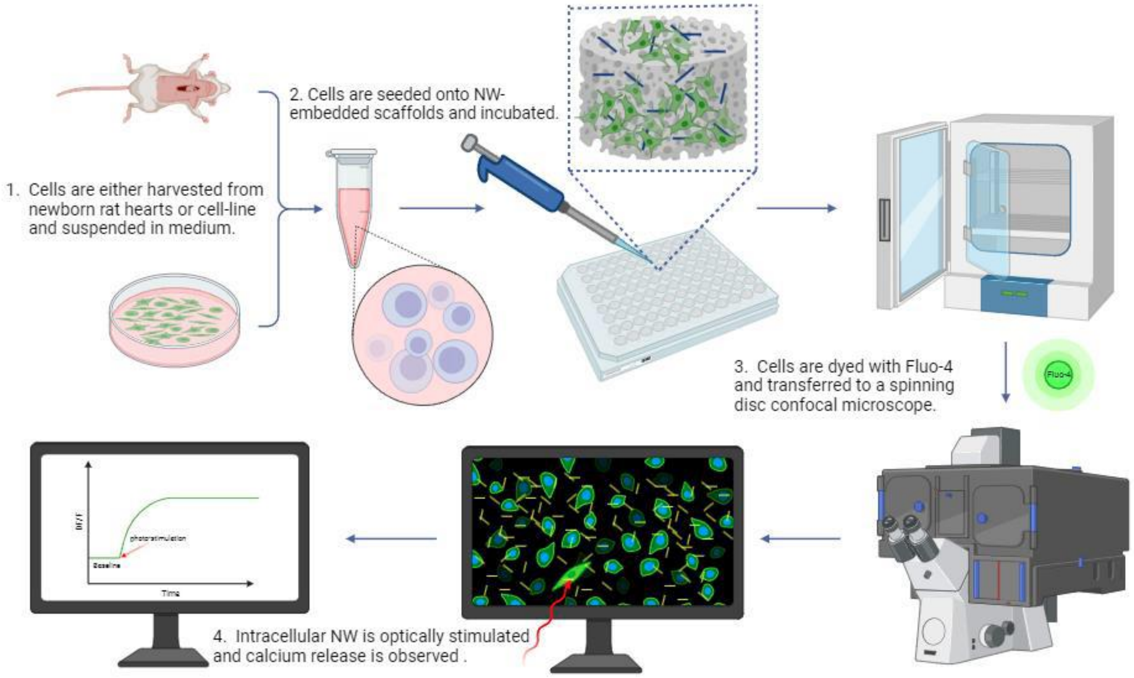

Figure S7: Illustration describing the experimental procedure of 3D intracellular photo-stimulation

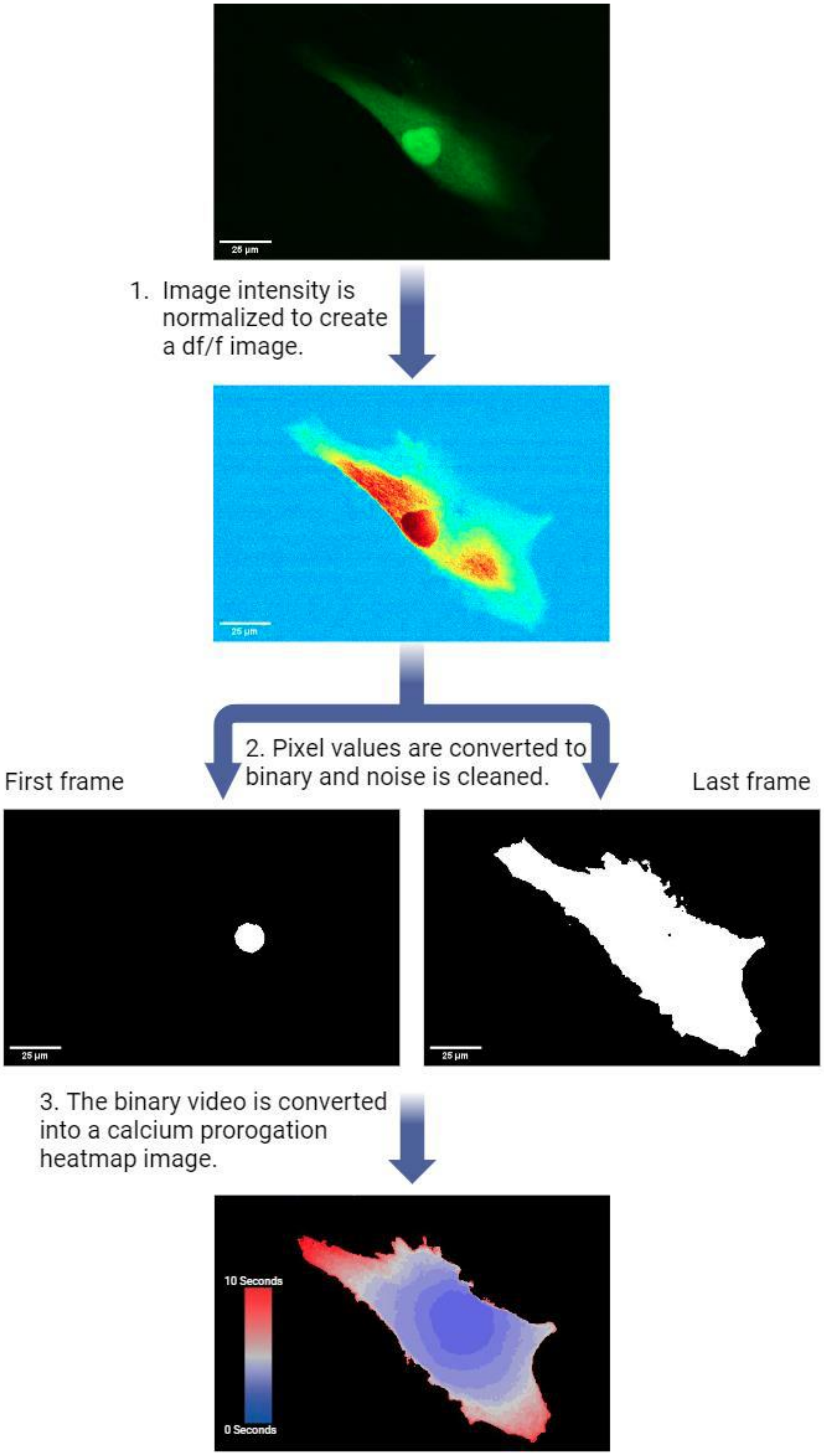

Figure S8: Image processing procedure of calcium propagation heatmaps
